# Sequential CD19/22 CAR T-cell immunotherapy following autologous stem cell transplantation for central nervous system lymphoma

**DOI:** 10.1038/s41408-021-00523-2

**Published:** 2021-07-15

**Authors:** Jiaying Wu, Fankai Meng, Yang Cao, Yicheng Zhang, Xiaojian Zhu, Na Wang, Jue Wang, Lifang Huang, Jianfeng Zhou, Yi Xiao

**Affiliations:** Department of Hematology, Tongji Hospital, Tongji Medical College, Huazhong University of Science and Technology, Wuhan, Hubei China

**Keywords:** Cancer immunotherapy, Lymphoma

## Abstract

Chimeric antigen receptor (CAR) T-cell immunotherapy following autologous stem cell transplantation (ASCT) is a promising method for refractory or relapsed multiple myeloma, but explicit data for central nervous system lymphoma (CNSL) are lacking. Here, we treated 13 CNSL patients with ASCT sequential CD19/22 CAR T-cell infusion and simultaneously evaluated the clinical efficacy and toxicity. The 13 CNSL patients analyzed included four primary CNSL and nine secondary CNSL patients. Patients 1 and 10, who had complete remission status before enrollment, maintained clinical efficacy without recurrence. Nine of the remaining 11 patients responded to our protocol with a median durable time of 14.03 months, and the overall response and complete remission rate were 81.81% and 54.55%, respectively. No patient suffered grades 3–4 cytokine-release syndrome (CRS), and only patient 10 experienced severe immune effector cell-associated neurotoxicity syndrome (ICANS). In addition, increases in serum ferritin and interleukin-6 levels were often accompanied by CRS and ICANS. After a median follow-up time of 14.20 months, the estimated 1-year progression-free survival and overall survival rates were 74.59% and 82.50%, respectively. Sequential CD19/22 CAR T-cell immunotherapy following ASCT as a novel method for CNSL appears to have encouraging long-term efficacy with relatively manageable side effects.

## Introductions

Central nervous system lymphoma (CNSL) is a lethal disorder with a poor prognosis, and it includes primary and secondary subtypes. Primary CNSL (PCNSL) is a rare subtype of extranodal non-Hodgkin lymphoma (NHL) that mainly involves the brain, eyes, leptomeninges and spinal cord without evidence of systemic NHL. Secondary CNSL (SCNSL) refers to systemic NHL with central nervous system (CNS) involvement or relapse [1, 2]. In recent years, aggressive high-dose methotrexate (HD-MTX)-based induction chemotherapy and autologous stem cell transplantation (ASCT) consolidation therapy have improved the clinical outcomes of CNSL [3, 4]. However, nearly three-fifths of PCNSLs appear to have refractory/relapsed (r/r) status, and fewer than 20% of patients with secondary CNS involvement or relapse of malignant lymphoma can achieve long-term survival [5–7]; therefore, more encouraging treatment strategies for CNSL need to be investigated.

Chimeric antigen receptor (CAR) T-cells serve as a novel immunotherapy method for r/r hematopoietic malignancies that result in a favorable clinical prognosis [8–10]; The “cocktail” treatment can effectively prevent tumor recurrence due to antigen escape [11–13]. However, the application of CAR T-cell immunotherapy in CNSL has been restricted due to concerns about the possibility of related severe cytokine-release syndrome (CRS) and immune effector cell-associated neurotoxicity syndrome (ICANS) [14]. Tu et al. [15] reported satisfactory clinical prognosis and controllable adverse events in a r/r primary CNS diffuse large-cell lymphoma (DLBCL) patient who received CD19/70 CAR T-cell infusion, indicating that CNSL was no longer an absolute contraindication for CAR T-cell immunotherapy. Another study conducted by our department also showed an objective response in CNSL patients who received CD19/ 22 CAR T-cell infusion, but only one complete remission (CR) case enrolled in another clinical trial of ASCT sequential CD19/22 CAR T-cell infusion achieved long-term progression/relapse-free survival; this finding demonstrated that separate CAR T-cell immunotherapy was effective but not long-lasting for CNSL, and the new model of CAR T-cell immunotherapy following ASCT provided a promising direction and option for the treatment of CNSL [16].

Here, we reported our findings for a total of 13 CNSL patients who underwent sequential CD19/22 CAR T-cell immunotherapy following ASCT based on preliminary experience. Our results indicate that sequential CD19/22 CAR T-cell immunotherapy following ASCT is a novel and promising method for CNSL patients to achieve long-term remission with manageable side effects.

## Materials and methods

### Study design

The present study was based on single-center, open-label, single-arm clinical trial (ChiCTR-OPN-16009847) data from January 1, 2019, to February 28, 2021, to observe the clinical efficacy and toxicity of sequential CD19/22 CAR T-cell immunotherapy following ASCT for CNSL. The trial was approved by the ethics committee of Tongji Hospital, Tongji Medical College, Huazhong University of Science and Technology. Informed consent was obtained by eligible patients and their families according to the Declaration of Helsinki.

### Therapy procedures

All enrolled patients received two separate apheresis procedures before the conditioning regimen, including disease-sensitive chemotherapy combined with granulocyte colony-stimulating factor (G-CSF)-stimulated autologous hematopoietic stem cell (HSC) collection and peripheral blood mononuclear cell (PBMC) apheresis for CAR T-cell manufacturing. CAR T-cell manufacturing-related quality control and analysis were completed by Wuhan Bio-Raid Biotechnology Co., Ltd., as previously described [10]. Patients could receive bridging therapy to further reduce the tumor burden before receiving the conditioning regimen at the discretion of professional physicians.

**Fig. 1:**
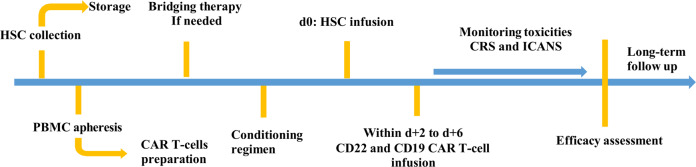
Therapy procedure. All eligible CNSL patients underwent two separate apheresis, and received conditioning regimen before HSC infusion on d0, two separate CAR T-cell products (CD22 and CD19 CAR T-cells) were infused within the range of 2–6 days (d + 2 to d + 6) after HSC infusion.

The conditioning regimen mainly included a thiotepa-based protocol and the BEAM protocol. The BEAM protocol included carmustine 300 mg/(m^2^·d) for day −6, etoposide 200 mg/(m^2^·d) from days −5 to −2, cytarabine 400 mg/(m^2^·d) from days −5 to −2, and melphalan 140 mg/(m^2^·d) for day −1; doxorubicin was given if needed. The thiotepa-based protocol included thiotepa 250 mg/(m^2^·d) from days −9 to −7, busulfan 3.2 mg/(kg·d) from days −6 to −4, and cyclophosphamide 60 mg/(kg·d) from days −3 to −2. Detailed dosages were adjusted at the physicians’ discretion according to the fundamental status and tolerance of patients. Two separate CAR T-cell products (CD19 and CD22 CAR T-cells) were infused within the range of 2 to 6 days (d + 2 to d + 6) after autologous HSC infusion (d0), and the infusion of CD22 CAR T-cell was usually one day prior to CD19 CAR T-cell considering the tolerance of patients. The therapy procedure was showed in Fig. 1.

### Efficacy and toxicity assessment

Neutrophil engraftment was defined as the first of three consecutive days with an absolute neutrophil count ≥0.5*10^9^/L. Response evaluation depended on the International PCNSL Collaborative Group Response Criteria and Lugano Response Criteria for B-Cell Lymphoma [2, 17]. CRS and ICANS were evaluated and graded according to the ASTCT Consensus Criteria [18, 19], and intervention therapies such as glucocorticoids or tocilizumab were given immediately according to the severity of CRS/ICANS and patient tolerance [20, 21]. Progression-free survival (PFS) was defined as the time from ASCT to progression, death or the last follow-up point, and overall survival (OS) was presented as the time from ASCT to death or the last visit [22]. Multiparameter flow cytometry was used to detect the CD19/22 CAR T-cell percentage in peripheral blood (PB) and cerebrospinal fluid (CSF). The expansion of CAR T-cells in vivo was determined by droplet digital polymerase chain reaction (ddPCR). Fluorescence in situ hybridization (FISH) was performed to detect the amplification or translocation of *MYC*, *BCL2*, and *BCL6*. Next-generation exome sequencing or HiSeq deep sequencing of 173 lymphoma-related genes was used for genetic mutation examination.

### Statistical analysis

All statistical analyses were performed using SPSS 25.0 and GraphPad Prism 8.0. Continuous variables are reported as medians and ranges, and categorical variables are reported as frequencies and percentages. The probability rates of PFS and OS were analyzed by the Kaplan–Meier method.

## Results

### Patient characteristics

In total, 13 CNSL patients with a median age of 42 years (range: 23–65 years) were enrolled in our study from January 1, 2019, to February 28, 2021. These patients comprised six (46.2%) males and seven (53.8%) females. Of the 13 patients, including four PCNSL and nine SCNSL patients, 10 were detected by FISH, and abnormal genetic factors mainly involved *BCL6*/*MYC* rearrangement/amplification and *TP53* deletion. *BCL6* and *MYC* rearrangements simultaneously occurred in case 8, which was diagnosed as “double hit” lymphoma. HiSeq deep sequencing was performed in nine patients, and with the exception of case 11, the remaining eight patients had positive pathogenic gene mutations, as shown in Table 1. Three of four patients with primary CNS DLBCL (cases 2, 6, 13) failed to completely respond to first-line therapy, and the remaining patient (case 10) achieved CR through a combination of surgery and HD-MTX-based chemotherapy but relapsed after long-term follow-up; this patient later reached CR status again after treatment with tumor-sensitive chemotherapy before enrolling in our study. Among the nine SCNSL patients, two (cases 1 and 4) had CNS involvement at the initial diagnosis, three (cases 7, 9, and 12) systemic NHL patients were refractory to chemotherapy and experienced CNS involvement in the course of treatment, and the remaining four (cases 3, 5, 8, and 11) systemic NHL patients previous with CR status developed CNS recurrence. At the time of enrollment in our study, patients 7 and 12 had coexisting CNS lesions and active lymph nodes. The details of previous therapy for all patients are shown in Supplement 1. More basic information is shown in Table 1.

**Table 1:** The clinical baseline characteristics of CNSL patients.

Case	Gender	Age (years)	Diagnosis	FISH	HiSeq deep Sequencing	Site of CNS disease	Systemic disease	Disease status
**1**	M	38	DLBCL IVB; GCB CNS involvement	Negative	*KMT2D*; *CREBBP*; *GNA13*; *ID3*	N	N	Chemotherapy refractory then CR after CD19/22 CAR T-cells treatment
**2**	M	55	Primary CNS DLBCL; non-GCB	*TP53* deletion; *BCL6* amplification;	*MYD88; TBL1XR1; TP53; TMSB4X; IGLL5; SMARCA4*	Left parietal lobe	N	PR
**3**	M	23	DLBCL IVA; non-GCB	*TP53* deletion	*NFKBIE; STAT6; TNFAIP3*; *SOCS1; IGLL5*	Right frontal lobe	N	CNS relapse
**4**	F	35	DLBCL IVA; GCB CNS involvement	*BCL6* rearrangement	NA	Bilateral temporal, occipital, and parietal lobe; callosum	N	CNS relapse
**5**	M	65	DLBCL IVB; non-GCB	*BCL6* amplification	*KMT2D; CD79B; MEF2B; TMSB4X*	Bilateral paraventricular	N	CNS relapse
**6**	F	39	Primary CNS DLBCL; non-GCB	NA	NA	Left eye, periocular tissue	N	PR
**7**	F	47	DLBCL IVA; non-GCB	*BCL6* rearrangement	NA	Left cauda hippocampus	Cervical LN	PD (CNS involvement)
**8**	F	32	DLBCL IVB; non-GCB	*BCL6/MYC* rearrangement	NA	Left basal ganglia and trigone of lateral ventricle	N	CNS relapse then PD
**9**	F	43	ILBCL IVB	NA	*NOTCH2*	Meninges	N	PD (CNS involvement) then SD
**10**	F	42	Primary CNS DLBCL; non-GCB	*TP53* deletion	*TP53; PIM1; ETV6; IRF4*	N	N	Relapse then CR
**11**	M	38	DLBCL IVA; non-GCB	NA	Negative	Cerebellum vermis and hemispheres	N	CNS relapse
**12**	M	55	DLBCL IVB; GCB	*BCL6* rearrangement	*CD70; FAS; DTX1, BCL10; KLF2; RRAGC; CDKN2A, IGLL5; EBF1*	CSF MRD: 7.2% tumor cells	Multiple LN	PD (CNS involvement)
**13**	F	47	Primary CNS DLBCL; non-GCB	*BCL6/MYC* amplification	*MYD88; TP53*	Right temporal lobe and thalamus	N	Relapse then PR

*M* male, *F* female, *CNS* central nervous system, *GCB* germinal center-like B-cell type, *DLBCL* diffuse large B-cell lymphoma, *ILBCL* intravascular large B-cell lymphoma, *NA* not available. *FISH* fluorescence in situ hybridization, *CR* complete remission, *PR* partial remission, *SD* stable disease, *PD* progression of disease, *MRD* minimal residual disease, *LN* lymph nodes.

### Response and survival

All patients received a conditioning regimen before HSC infusion; 38.5% (5/13) of sufferers used a thiotepa-based protocol, and 61.5% (8/13) chose the BEAM regimen. The median dosage of CD34^+^ cell infusion was 8.4 (2.0-33.4) *10^6^/kg, the CD22 and CD19 CAR T-cells were separately infused, and the median cell number were 4.1 (2.6-8.4) *10^6^/kg and 4.3 (2.0-9.2) *10^6^/kg, respectively. Enrolled patients achieved successful neutrophil engraftment with a median period of 13 days (range: 9-20 days) (Table 2).

**Table 2:** The treatment and efficacy of CNSL patients received CD19/22 CAR T-cells infusion following ASCT.

Case	Conditioning regimen	CD34^+^ cells (*10^6^/kg)	CD22 CAR T-cells (*10^6^/kg)	CD19 CAR T-cells (*10^6^/kg)	Neutrophil engraftment (d)	CRS grade/symptoms	ICANS grade/symptoms	Best response (duration: months)	Survival^a^
1	Dox + BEAM	2.0	3.7	4.4	12	1 (Fever)	N	CR (24.17)	Yes
2	TBC	32.4	4.0	4.0	13	N	N	CR (19.27)	Yes
3	Dox + BEAM	16.4	4.1	6.0	9	1 (Fever)	N	CR (18.53)	Yes
4	TBC	13.1	4.0	4.0	9	2 (Fever; Hypotension)	N	CR (17.43)	Yes
5	Dox + BEAM	8.1	8.4	9.2	20	1 (Fever)	N	CR (16.03)	Yes
6	Dox + BEAM	2.9	4.3	3.6	19	1 (Fever)	N	CR (14.03)	Yes
7	TBC	8.4	2.6	2.0	12	1 (Fever)	1 (Apathetic; Memory impairment)	PD (0.40)	No
8	TBC	33.4	5.0	5.0	11	1 (Fever)	N	PD (0.60)	No
9	Dox + BEAM	3.2	2.7	4.3	15	1 (Fever)	N	CR (11.17)	Yes
10	TBCF	5.7	5.0	5.0	15	1 (Fever)	3 (Delirious; Disoriented)	CR (4.67)	Yes
11	TBCF	8.6	5.8	2.0	13	2 (Fever; Hypoxemia)	N	PR (0.90)	Yes
12	TBC	4.0	2.8	2.0	17	N	1 (Lethargy)	PR (0.33)	Yes
13	TBC	19.3	5.0	5.0	12	1 (Fever)	N	PR (0.23)	Yes

*TBC* *±* *F* thiotepa, busulfan, cyclophosphamide ± Flu, *BEAM* carmustine, etoposide, cytarabine, melphalan, *CAR* chimeric antigen receptor, *Neu* neutrophil, *CRS* cytokine-release syndrome, *ICANS* immune effector cell-associated neurotoxicity syndrome.

^a^Follow-up to March 15, 2021 or death.

**Fig. 2:**
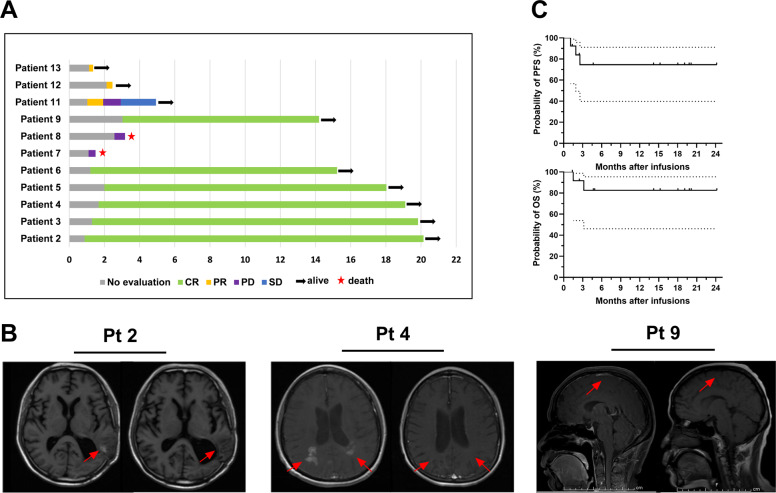
Descriptions of the clinical results. **A** The clinical outcomes (excluded patient 1 and 10), the last visit day was March 15, 2020. **B** Representative MRI imaging before (left) and after (right) therapy. **C** The probability of PFS and OS. The median PFS and OS of patients were undefined, the 1-year estimated PFS and OS rate were 74.59% (95% CI: 39.76–91.10%) and 82.50% (95% CI: 46.10–95.33%), respectively.

Among the enrolled 13 patients, case 1 achieved CR status by receiving two separate CAR T-cell products (CD22 and CD19 CAR T-cells) “cocktail” treatment before enrollment, and case 10 also was MRI negative, which attributed to tumor-sensitive chemotherapy, both of the above patients received our protocol therapy for further consolidation, and maintained clinical efficacy without recurrence until the last visit. Nine of the remaining 11 patients responded to CD22/19 CAR T-cell immunotherapy following ASCT within 3 months, including six who achieved CR and three who achieved partial remission (PR). The overall response rate (ORR) and complete remission rate (CRR) were 81.81% and 54.55%, respectively. Patient 11 developed disease progression after achieving PR status in the first efficacy assessment. The median durable time for the nine responsive patients was 14.03 months (range: 0.23–19.27 months); 18.18% (2/11) of patients (cases 7 and 8) did not respond to the current treatment protocol and maintained progressive disease (PD) status (Fig. 2A). MRI images for three representative patients before therapy and at the time of best response are shown in Fig. 2B.

As of March 15, 2021, the median follow-up time of all enrolled patients was 14.20 months (range: 1.37–24.17 months), and only two systemic NHL patients with CNS involvement/relapse (cases 7 and 8) refused further treatment due to multiple virus infection and/or rapid disease progression. Both died shortly after leaving hospital, with a median survival time of 2.33 months (range: 1.5–3.17 months). The median PFS and OS after ASCT and CAR T-cell infusion were undefined, and the estimated 1-year PFS and OS rates were 74.59% (95% CI: 39.76–91.10%) and 82.50% (95% CI: 46.10–95.33%), respectively (Fig. 2C).

### Side effects

**Fig. 3:**
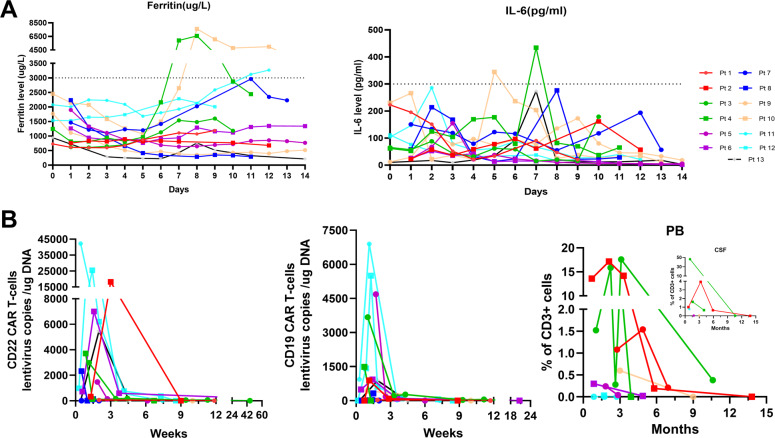
The variation of inflammatory markers and CAR T-cell kinetics. **A** The serum ferritin and IL-6 level of patients. Increases in serum ferritin and IL-6 level were often accompanied by CRS and ICANS. **B** Lentivirus copies in vivo and percentage of CAR T-cells in CD3 + cells. The median peak number of CD19 and CD22 CAR T-cells lentivirus copies were 911 and 3346 copies/µg DNA, and the peak percentage of CAR T-cells in CD3 + cells in CSF and PB were 46.38% and 17.58%, respectively.

After CAR T-cell infusion, 11 (84.62%) patients (except cases 2 and 12) had low-grade CRS. Nine had fever over 38 °C and were assessed as having grade 1 CRS, and two exhibited grade 2 CRS and presented with fever and hypotension or hypoxemia; no patient suffered grades 3–4 CRS. In addition, case 7 presented with apathetic and mild memory impairment (ICE score: 8 points) and case 12 with lethargy (ICE score: 9 points) both were assessed as grade 1 ICANS. Patient 10, who appeared delirious and disoriented (ICE score: 1 point), was diagnosed with grade 3 ICANS. The cumulative incidence of ICANS was 27.27%. Grade 1 CRS cases were reversed after timely administration of broad-spectrum antibiotics, while patients with ICANS or grade 2 CRS were recovered by simultaneously combing with intravenous methylprednisolone injection, no patient had residual neurological impairment. Other side effects included viremia (4/13) and upper respiratory infection (1/13); one (case 7) patient refused further therapy because of multiple virus infection (CMV, JCV, BKV, TTV) and disease progression, and others were supported by effective anti-infection treatment. As shown in Fig. 3A, the developed ferritin index varied, increasing from the baseline level except for cases 9 and 13, as did the IL-6 level, excluding cases 1 and 12. Ferritin and IL-6 reached the peak levels (ferritin: 2235 µg/L; IL-6: 194 pg/mL) on the median 8th and 7th day after the first CAR T-cell infusion, respectively. In addition, increases in serum ferritin and IL-6 levels were often accompanied by CRS and ICANS, and patients who experienced grade 2 CRS or grade 3 ICANS had peak serum ferritin and IL-6 levels greater than 3000 µg/L and 300 pg/mL, respectively.

### CAR T-cell kinetics

Except for patient 10, the remaining 12 patients underwent ddPCR testing for absolute quantification of CAR gene expression to better understand the expansion of CAR T-cells in vivo. As shown in Fig. 3B, the median peak numbers of CD19 and CD22 CAR T-cells lentivirus copies in vivo were 911 (range: 33–6900) and 3346 (range: 57–42188) copies/µg DNA, respectively, and achieved peak levels with median times of 1.36 (range: 0.72–1.86) and 1.46 (range: 0.43–3.00) weeks after CAR T-cell infusion, respectively. Interestingly, the lentivirus copies of CD19 and CD22 CAR T-cells in all patients except patient 8 (who suffered early death without explicit data) were unabiding, only with median durable times of 8.43 (1.29–20.72) and 8.57 (2.00–45.43) weeks, respectively. The CAR T-cells percentage in CD3 + cells was measured by flow cytometry; only case 2 received regular detection monthly for the first 3 months after CAR T-cells infusion, followed by evaluations at ~6 and 12 months, and the peak percentages of CAR T-cells in CD3 + cells in the CSF and PB were 46.38% and 17.58%, respectively.

## Discussion

Recently, the “living” drug of CAR T-cell infusion following ASCT has been demonstrated to be relatively effective and safe for r/r multiple myeloma and B-cell NHL [22–24]. Several studies have also reported the clinical efficacy of CNSL receiving separate CAR T-cell immunotherapy, but limited data on ASCT sequential CAR T-cell infusion have been reported [15, 25, 26]. Herein, we reported the clinical efficacy and toxicities of 13 CNSL patients (four PCNSL and nine SCNSL) received sequential CD19/22 CAR T-cell immunotherapy following ASCT, no patient suffered grades 3-4 CRS, and only one patient experienced severe immune ICANS. The ORR and CRR were 81.81% and 54.55%, and the estimated 1-year PFS and OS rates were 74.59% and 82.50%, respectively. Our preliminary results showed that CNSL patients who received sequential CD19/22 CAR T-cell immunotherapy following ASCT achieved long-term remission without serious toxicities.

In our present study, two patients with CR status were voluntarily enrolled in our trial for further consolidation considering the aggressive progression of CNSL, and both achieved long-term disease/relapse-free survival, indicating that ASCT sequential CAR T-cell infusion can have a long-term consolidation effect for r/r high-risk CNSL patients. In addition, nine of the remaining 11 patients responded within 3 months after treatment, and the duration of recurrence-free CR was more than a year in five of six patients. This is inconsistent with previous reports that some patients with CNSL who received CAR T-cell infusion or ASCT can obtain an objective clinical response but not an enduring response [6, 16]. The main reasons for this difference are as follows. First, a conditioning regimen and ASCT can minimize the tumor burden and disease activity and deeply deplete lymphocytes that inhibit CAR T-cell function [22]. Second, the ability of HSC to improve the complex and unique tumor immunosuppressive microenvironment of the CNS may be beneficial to reduce the possibility of relapse [27]. Last, the target killing effect of CAR T-cells to purify the graft can effectively avoid the recurrence caused by product contamination [28, 29].

However, several (2/13) patients did not respond to our treatment and died of rapid disease progression, with a median survival time of 2.33 months; however, no deaths were attributed to CAR T-cell products. The two unresponsive patients were both in PD status before our enrollment, and the lack of response may be associated with rapid growth of tumor cells counteracting the effects of HSCs and CD19/22 CAR T-cells. A significant proportion of aggressive disease patients lose the opportunity for CAR T-cell infusion due to rapid progression or clinical deterioration, so other preferential therapies should be given to maximize killing tumor cell populations to improve the therapeutic effect before ASCT sequential CAR T-cell immunotherapy, especially for patients with PD or stable disease status accompanied by high tumor burden [22]. Patient 8, with high-risk double hit (*BCL6* and *MYC* rearrangement) disease, belongs to a population with historically poor prognosis, which may also result in a nonresponsive outcome [30, 31].

All patients reached peak levels of lentiviral copies of CD19/22 CAR T-cells within 2 weeks, which was consistent with previous reports on hematological malignancies receiving CAR T-cell therapy [32, 33]; however, another attractive phenomenon was the undetectable lentiviral copies of CD19/22 CAR T-cells in vivo within a median time of <3 months after infusion. Even in patients with a durable response, the sustainable effects did not appear to require prolonged expansion and persistence of functional CAR T-cells. The mechanism of this phenomenon still needs to be investigated. We speculate that it may be related to the lack of effective antigen stimulation in durable remission patients based on the past conclusion that low CD22 expression impaired in vivo CD22 CAR T-cell persistence [34]. In addition, a small portion of effector CAR T-cells may have been induced and transformed into durable memory CAR T-cells to promptly exert the antitumor effect upon recurrence [35], but the level in the blood is lower than the detectable threshold of present quantification technology. More sensitive and accurate detection techniques for the quantification of transgenic CAR T-cells may contribute to increasing the number of positive results [36, 37].

CRS and ICANS, as the most common CAR T-cell-associated side effects, should be closely monitored, and previous studies on CAR T-cell immunotherapy have excluded CNSL patients due to concerns about potentially fatal ICANS [14, 25]. In our present study, most patients developed fever (≥38 °C) after CAR T-cell infusion and were assessed as having grade 1 CRS. No patient experienced grades 3–4 CRS, and only one patient suffered grade 3 severe ICANS. For all patients, these symptoms were reversible by timely therapy, and no patient died of severe side toxicities. Previously, a multicenter, phase 2 trial [38] conducted by Neelapu et al. in 111 DLBCL patients who received CD19 CAR T-cell therapy indicated that grades 3 or higher CRS and ICANS occurred in 13% and 28% of recipients, respectively. No obvious difference or elevation was observed in our current research, occasionally, the results were even better than those of separate CAR T-cell therapy, which was also consistent with other earlier related reports, including those related to ASCT sequential CAR T-cell infusion for other hematological malignancies [10, 24, 25, 38, 39]. The acceptable CRS and ICANS rates may be attributed to the use of an enhanced conditioning regimen and ASCT before CAR T-cell infusion to minimize the tumor burden and myeloid cells, which have been confirmed to be related to the occurrence and severity of CRS and ICANS [39–41]; moreover, myeloid cell-derived cytokines (IL-1, IL-6, GM-CSF, etc.) were the major sources of CRS after CAR T-cell immunotherapy [42–44]. Of course, the manageable side effects can also be attributed to the development of supportive therapy and CAR T-cell manufacturing-related technology. Taken together, our research further indicated that CNSL no longer has to be considered a contraindication to CAR T-cell immunotherapy and that ASCT sequential CD19/22 CAR T-cell therapy for CNSL patients proved to have low toxicity.

Meanwhile, we detected the daily levels of IL-6 and ferritin during hospitalization and attempted to analyze the relationship between the levels of serum cytokines and inflammatory markers and the occurrence of CRS and ICANS. Consistent with earlier studies, we observed that elevated serum ferritin and IL-6 levels were often accompanied by CRS and ICANS; moreover, the degree was positively related to the ferritin and IL-6 levels [38, 45–47]. The relationships among CRS, ICANS and other cytokines (such as IL-10 and interferon-γ) and indicators (such as angiopoietin and von Willebrand factor) need to be monitored and analyzed in future studies.

In general, our preliminary experience demonstrated that sequential CD19/22 CAR T-cell immunotherapy following ASCT acted as a novel therapy for CNSL and appears to have encouraging long-term efficacy with relatively manageable side effects. However, there are some limitations in our present study. The number of cases for analysis was small, and the follow-up time for some patients was relatively short. It is necessary to proceed with multicenter prospective research to further clarify the clinical efficacy and safety of CD19/22 CAR T-cell immunotherapy following ASCT for CNSL. Meanwhile, a clinical trial comparing ASCT and CAR T-cell therapy with ASCT or CAR T-cells alone in treating CNSL patients should be conducted.

## Supplementary information

Supplement 1. Previous therapy of all patients.

Reproducibility checklist
