# Supplementary material for: Sequential CD19/22 CAR T-cell immunotherapy following autologous stem cell transplantation for central nervous system lymphoma
Source: Blood Cancer J. 2021 Jul 15;11(7):131. doi: 10.1038/s41408-021-00523-2 (PMC8282870; doi:10.1038/s41408-021-00523-2)
Supplement: Supplementary file 1 — Supplement 1. Previous therapy of all patients. [file 41408_2021_523_MOESM1_ESM.docx]

**Supplement 1. Previous therapy of CNSL patients**

| **Case** | **Previous therapy** |
| --- | --- |
| **1** | Chemotherapy (NA^#^); CD22/19 CAR T therapy (CR) |
| **2** | Surgery; HD-MTX+R+ Temozolomide×1(PD); HD-MTX+R+ Ibutinib ×4 (PR);  R+ Doxorubicin+ Lenalidomide+ Ibutinib ×1 |
| **3** | R-CHOP ×8(CR then CNS relapse 3 months later); HD-MTX+R ×2; Hyper CVAD B ×1 |
| **4** | R-CHOP ×1+ Radiotherapy (PD); R-MAD ×2 +MAD ×5 +RMD ×1 (CR);  HD-MTX+R+ PD-1 inhibitor ×1(CNS relapse); HD-MTX+R ×1 |
| **5** | R-CHOP ×4 (PR); R-CHOP ×4 (CR); R×2 (CR then relapse 14 months later); R-DHAP ×4 (CR);  Chidamide (CNS relapse 9 months later); HD-MTX+ Temozolomide ×1; MA ×1 |
| **6** | R-EPOCH ×4 (SD)；R-DICE ×2 (SD); IMRT ×10 (PR); R-DHAP ×1 |
| **7** | R-CHOP (PD: CNS involvement); R-DHAP ×1 |
| **8** | R-CHOP ×8 (CR then CNS relapse); HD-MTX+ Temozolomide ×4 (PD); Hyper CVAD B ×1 |
| **9** | R-DEP ×2 + R-CHOP ×2 (PR); CHOP ×1 (CNS involvement); HD-MTX+ R ×1 (PD);  HD-MTX+ Cytarabine ×4 (PR then PD); Lenalidomide+ Temozolomide ×3 (SD) |
| **10** | Surgery; HD-MTX+ DEX ×4; WBRT (CR Then CNS relapse 16 months later); HD-MTX+R +Ara-C ×2 (CR); R-CYVE ×1 |
| **11** | R-CHOP ×4 (CR then CNS relapse 4 months later); CYVE ×1 |
| **12** | R-CHOP ×5(PD: CNS involvement); R-DA-ECHOP ×1; R+ Gemox ×1(PD) |
| **13** | Surgery; HD-MTX+R×2(CR); HD-MTX+R + Temozolomide×1 (CR then relapse);  Zebutinib+ R-hyper CVAD B ×1 (PR) |
